# Impact of leachate on groundwater pollution due to non-engineered municipal solid waste landfill sites of erode city, Tamil Nadu, India

**DOI:** 10.1186/1735-2746-9-35

**Published:** 2012-12-27

**Authors:** Rajkumar Nagarajan, Subramani Thirumalaisamy, Elango Lakshumanan

**Affiliations:** 1 Department of Civil Engineering, Kongu Engineering College, Perundurai, Tamil Nadu, India; 2 Department of Geology, Anna University, Chennai, Tamil Nadu, India

**Keywords:** Solid waste management, Groundwater contamination, Landfill, Leachate

## Abstract

Leachate and groundwater samples were collected from Vendipalayam, Semur and Vairapalayam landfill sites in Erode city, Tamil Nadu, India, to study the possible impact of leachate percolation on groundwater quality. Concentrations of various physicochemical parameters including heavy metals (Cd, Cr, Cu, Fe, Ni, Pb, Fe and Zn) were determined in leachate samples and are reported. The concentrations of Cl^-^, NO_3_^-^, SO_4_^2-^, NH_4_^+^ were found to be in considerable levels in the groundwater samples particularly near to the landfill sites, likely indicating that groundwater quality is being significantly affected by leachate percolation. Further they were proved to be the tracers for groundwater contamination near Semur and Vendipalayam dumpyards. The presence of contaminants in groundwater particularly near the landfill sites warns its quality and thus renders the associated aquifer unreliable for domestic water supply and other uses. Although some remedial measures are suggested to reduce further groundwater contamination via leachate percolation, the present study demands for the proper management of waste in Erode city.

## Introduction

Landfills have been identified as one of the major threats to groundwater resources (Fatta *et al.,*[1]) not only in India but throughout the world (United States Environmental Protection Agency US EPA [2]). More than 90% of the Municipal Solid Waste (MSW) generated in India is directly dumped on land in an unsatisfactory manner (Chatterjee, [3]). The solid waste placed in landfills or open dumps are subjected to either groundwater underflow or infiltration from precipitation or any other possibility of infiltration of water. During rainfall, the dumped solid wastes receivers water and the by-products of its decomposition move into the water through the waste deposition. The liquid containing innumerable organic and inorganic compounds is called 'leachate'. This leachate accumulates at the bottom of the landfill and percolates through the soil and reaches the groundwater (Mor et al. [4]).

Areas near landfills have a greater possibility of groundwater contamination because of the potential pollution source of leachate originating from the nearby dumping site. Such contamination of groundwater results in a substantial risk to local groundwater resource user and to the natural environment. The impact of landfill leachate on the surface and groundwater has given rise to a number of studies in recent years and gained major importance due to drastic increase in population (Saarela, [5]). There are many approaches that can be used to assess the groundwater and surface water contamination. It can be assessed either by the experimental determination of the impurities or their estimation through mathematical modeling (Moo-Young *et al.,*[6]).

In the present study, the impact of leachate percolation on groundwater quality was estimated from an unlined landfill site at Vendipalayam, Semur and Vairapalayam of Erode District, Tamil Nadu, India. Various physicochemical parameters including heavy metals were analyzed in the leachate and in groundwater samples to understand the possible link of groundwater contamination. The effect of depth and distance of landfill from groundwater sources were also studied and some remedial measures were discussed to reduce further contamination of groundwater.

## Materials and methods

### Study area details

Erode city is the head quarters of Erode District, Tamil Nadu, India which sprawls over 120 km^2^ and lies between 11° 17' N and 11° 23' N latitudes and 77° 40' E and 77° 46' E longitudes (Fig-01). The average altitude of the region is about 172 m above the mean sea level. It is situated at the center of the South Indian Peninsula, about 400 kilometres southwest from the state capital Chennai and on the banks of the rivers Cauvery and Bhavani. It is located on the western bank of the Cauvery River, while its twin city, Pallipalayam, is on the eastern bank of the river. Erode in general is characterized with scanty rainfall and a dry climate.

Erode has moderate-dry weather throughout except during the monsoon seasons. It also experiences heavy rains primarily during the periods of monsoon with an average annual rainfall of 700 mm. The depth of groundwater table in Erode city varies from 1 to 15 m with respect to ground level.

Erode city, with a population of over 1,50,000 is estimated to generate about 75 tonnes of garbage daily. The daily per capita generation of solid waste in Erode city ranges from 100 g to 500 g, which depends upon the economic status of the community involved (Mor *et al.*, [4]). The important categories of MSW in the city includes waste from household, industries and medical establishments. The solid waste generation rate also varies from 0.66 kg/capita /day to 0.44 kg/capita/day in rural areas (Ogwueleka, [7]).

**Figure 1:**
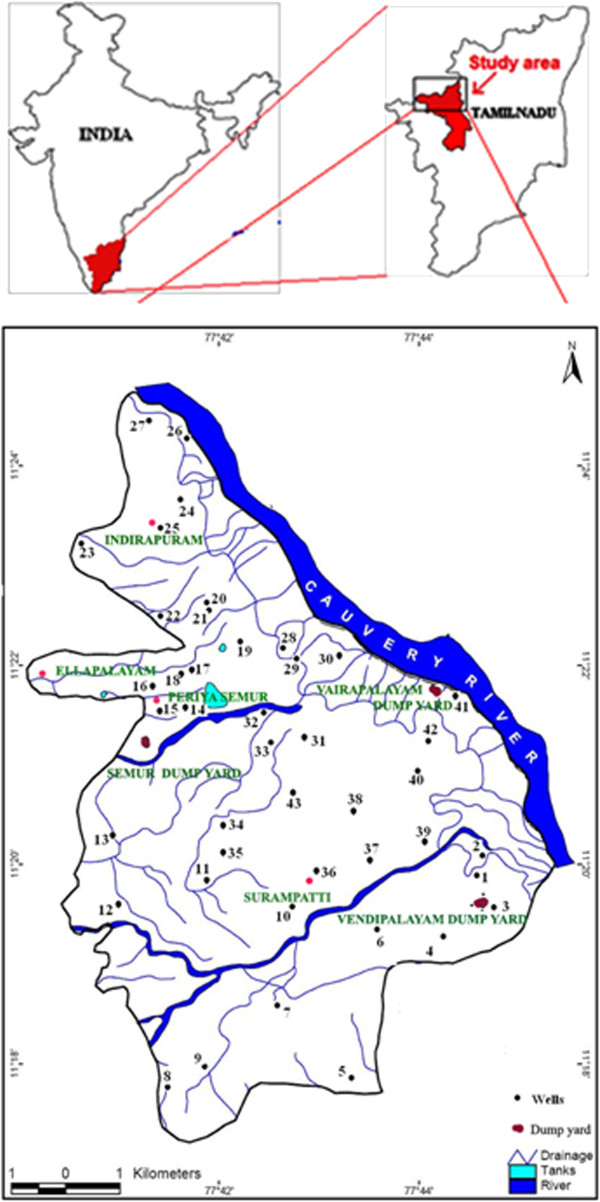
Study area map showing locations of observation wells and solid waste dump yards.

The earliest landfill was started in Erode in 1963 near Vendipalayam at a distance of 1 km from the city centre. Three landfill sites within the city premises are filled and closed. All of them are unlined and non-engineered landfill sites. At present three functioning landfill sites are located at Vendipalayam, Semur and Vairapalayam (Figure 1). These sites are spread over an area of about 1,12000 m^2^. None of their bases are lined, which may result in continuous groundwater contamination. These sites have not been designed systematically before being used for disposal/dumping of waste. Furthermore, no environmental impact assessment (EIA) has been carried out prior to selection of these sites.

### Vendipalayam landfill site

The Vendipalayam landfill site started in the year 1963 is still in use. It spreads over an area of approximately 65000 m^2^ and is situated in the eastern part of the city. On an average, 45 T/day of waste is dumped and the waste fill height varies from 12 m to 15 m. It contains about 367 pits. The size of the each pit is 12 m × 2.5 m × 1.5m. It is located at the close proximity of the living communities. The waste dumped at this site mainly includes domestic waste such as kitchen waste, paper, plastic, glass, cardboard and clothes. Construction and demolition waste consisting of sand, bricks and concrete block are also dumped (Mor *et al.*, [4]). Further, wastes from the adjacent vegetable market, fish market and slaughterhouse are also dumped here.

The site is a non-engineered and low lying open dump yard which looks like a huge heap of waste up to a height of 12–15 m. Trucks and separate vehicles from different parts of the city collect and bring the waste to this site and dump in irregular arrangement. The waste is dumped as such without segregation, except the rag pickers who rummage through the garbage and help in segregating it. They generally collect glass material, plastic and metals and sell this to the recycling units (Aggarwal *et al.,*[8]). At this landfill site one bore well is operational, which is used for washing of removal vehicles and maintenance of heavy earth moving equipments.

### Semur landfill site

Semur landfill site started in the year 2001, is currently in use. The area of the site is approximately 32,000 m^2^. On an average 15 to 20 T/day of wastes are dumped in the site. Mostly the wastes in and around Semur panchayat are dumped at this site. The approximate population contributing wastes to this site is around 55,000. The waste fill height at this site varies from 9 m to 11 m. The waste dumped at Semur site includes kitchen wastes, papers, wood, plastics, cardboard and clothes. Semur landfill site is also a non-engineered open dumping site, which causes various problems to the living community near the site. Trucks from various parts of the Semur municipality collect the waste materials and dump it at the site without any regular arrangement. Semur is located within the corporation limit at a distance of 7 km from the Erode city center.

### Vairapalayam landfill site

Vairapalayam is a small village located on the right bank of Cauvery River, which forms the northern boundary of Erode city and falls within the Erode corporation limit. Landfill site was started at Vairapalayam in the year 2001, which is also currently in use. This is also a non-engineered municipal solid waste dumping site, which is located in the low lying region of the city. The area of the site is approximately 15000 m^2^. On an average, around 20 T/day of garbage is dumped at this site. The site remains submerged in the river water during high flood season, which may also cause pollution to the surface water. The dumping site is located at a distance of 6 km from city center. Wastes collected from the northern part of the city are transported to this landfill site by trucks.

## Materials and methods

### Sampling of leachate and groundwater

In an effort to study the extent of the groundwater contamination in Erode city, 43 observation wells were selected to collect groundwater samples (Figure 1). The geomorphological study of the region indicates that most of the area where the landfill sites are located is found to have deep pediments. The region also consists shallow and buried pediments in other parts. The groundwater samples were collected during February after the extraction of water from open wells. The field testing kit was taken to the sampling sites and the important insitu parameters such as temperature, electrical conductivity (EC), pH, salinity and dissolved oxygen (DO) were tested at the site itself. Since the landfill sites are not equipped with leachate collectors, the leachate at the base of the landfills were sampled randomly at three different locations in each dumping site (Shuokr Qarani *et al.,*[9]).

### Analytical methods

Samples were immediately transferred to the laboratory and were stored in refrigerator at 4°C. The sampling wells were selected based on the availability of the wells around the landfill sites and to represent the whole area (Buket Yenigul *et al.,*[10]). The analysis was started without any delay using the standard procedure prescribed by the American Public Health Association (APHA, [11]). All the samples were analyzed for selected relevant physico-chemical parameters and heavy metals. Various physico-chemical parameters examined in groundwater samples include, total dissolved solids (TDS), total alkalinity (TA), total hardness (TH), calcium (Ca^2+^), magnesium (Mg^2+^), sodium (Na^+^), potassium (K^+^), ammonia (NH_4_^+^), chloride (Cl^-^), fluoride (F^-^), sulphate (SO_4_^2-^) and nitrate (NO_3_^-^). Estimation of COD for leachate was done by reflux titrimetry, while BOD was calculated using oxygen determination by Winkler titration. TA, TH, Ca^2+^, Mg^2+^ and Cl^-^ were estimated by titration, and Na^+^ and K^+^ were estimated using flame photometer (Systronic-128).

Fluoride was estimated by SPANDS, while SO_4_^2-^, NO_3_^-^, NO_2_^-^ and NH_4_^+^ were determined using UV/VIS spectrophotometer. The concentrations of cadmium (Cd), copper (Cu), chromium (Cr), iron (Fe), nickel (Ni), lead (Pb) and zinc (Zn) were determined using a SpectrAA-20 (Varian) Atomic Absorption Spectrometer (AAS). The limit of detection (LOD) of these elements was 0.02, 0.03, 0.06, 0.03, 0.1, 0.1, and 0.01 mg/L, respectively. The depth of groundwater table was also measured in each well using water level recorder. It varied between 1 and 15 m with respect to the ground surface (Table 1). All the experiments were carried out in triplicate and the results were found reproducible within ±3% error. The data were statistically analyzed by setting up and calculating a correlation matrix for the various parameters using Statistical Package for Social Sciences (SPSS**)**.

**Table 1:** Range of depth of groundwater table in observation wells

**S.No**	**Range of depth of groundwater levels with respect to ground surface**	**Representing wells**
01.	1-5 m	1-3,5,7-9,11-19,21,22,24-30,34,35,39-41
02.	5-10 m	4,6,20,23,32,33,36-38,42
03.	10-15 m	10,31,43

## Results

### Leachate characteristics

Physico-chemical charascteristics of the leachate depend primarily upon the waste composition and water content in total waste (Mohan and Gandhimathi [12]). The characteristics of the leachate samples collected from the Vendipalayam, Semur and Vairapalayam landfill sites are presented in Table 2. The pH value of lechate of Vendipalayam landfill site was 6.9. The TDS (25514 mg/L), BOD (17,552 mg/L) and COD (25,102 mg/L) values were high in the leachate samples of Vendipalayam landfill during February. Among the nitrogenous compounds, ammonia nitrogen (1932 mg/L) was present in high concentration. A high concentration of NO_3_^-^ (361 mg/L) was also observed in the leachate samples.

**Table 2:** Physico-chemical characteristics of leachates at various landfill sites

**Parameters**	Concentrations* at Vendipalayam Landfill Site	Concentrations* at Semur Landfill Site	Concentrations* at Vairapalayam Landfill Site
pH	6.9	6.9	6.7
TDS	25514	22961	24123
COD	25102	22148	23900
BOD	17552	15478	15691
Na^+^	532	462	393
K^+^	1392	1241	1136
NH_4_^+^	1932	1622	2231
NO_2_^-^	Nil	Nil	Nil
NO_3_^-^	361	321	352
Phenol	0.02	0.01	316
Cd	0.05	0.02	0.05
Cr	0.23	0.28	0.14
Cu	0.89	0.71	0.71
Fe	63.41	58.91	58.40
Ni	0.38	0.31	0.31
Pb	1.10	1.20	1.31
Zn	2.10	1.29	1.29

*All in mg/L except pH and EC (μS/cm).

The high level of Fe (63.41 mg/L) in the leachate sample indicates that iron and steel scrap are also dumped in the landfill at a larger quantity (Bendz *et al.,*[13]). The presence of Zn (2.10 mg/L) in the leachate shows that the landfill receives waste from batteries and fluorescent lamps. The presence of Pb (1.10 mg/L) was also detected in the leachate samples but the concentration was comparatively lower. Cr (0.23 mg/L), Cu (0.89 mg/L) and Ni (0.38 mg/L) were also present in the leachate samples. A variety of waste is dumped at the landfill sites, which likely indicates the origin of Zn, Pb, Cr, Cu and Ni in leachate (Christensen *et al.,*[14]).

The TDS value in the Semur landfill site was found to be 22961 mg/L. The BOD and COD of the leachate samples were 15478 mg/L and 22148 mg/ L. The presence of high BOD and COD values indicates the organic strength of the sample taken for analysis (Zgajnar Gotvajn *et al.,*[15]). The Fe content in the leachate sample of Semur landfill site was 58.91 mg/L. The presence of various heavy metals was identified in the leachate sample of Semur landfill site.

High levels of BOD (15691 mg/L) and COD (23900 mg/ L) were found in the leachate samples of Vairapalayam landfill site. The ammonia nitrogen of the sample from Vairapalayam landfill site is found to be 2231 mg/L. The samples were also tested for the presence of trace metals like Cd, Cr, Cu, Fe, Ni, Pb and Zn, and the concentrations of these metals was 0.05, 0.14, 0.71, 58.40, 0.31, 1.31 and 1.29 mg/L respectively. TDS (24123 mg/L) was also very high in the leachate samples of Vairapalayam landfill site.

### Groundwater chemistry

#### Physicochemical characteristics

The groundwater of the study area is mainly used for domestic purposes. It is also used for irrigation purposes in many places. Therefore it is essential to assess the suitability of groundwater for drinking and irrigation purposes. Table 3 presents the summary of analytical results of the groundwater samples and the comparison of groundwater quality parameters with Bureau of Indian Standard (Bureau of Indian Standards BIS [16]) and World Health Organization standard (WHO, [17]). The comparison highlights that some of the chemical constituents exceed the permissible limit for drinking. The pH variation indicates that the groundwater of the study area is slightly alkaline in nature. EC values in the study area ranged between 410 and 3830 μmhos/ cm. It was noticed that groundwater samples collected near the landfill sites contain more soluble salts. Most of the groundwater samples in the study area were within the maximum permissible limit for drinking as per the BIS and WHO standards, except some samples. Groundwater chemistry of a region may also be influenced by complex contamination sources and geochemical processes (Subramani *et al.*, [18]). The contamination levels are high in the wells near to the landfill sites.

**Table 3:** Comparison of groundwater quality parameters with Indian (BIS) and International (WHO) standards

**Parameters**	**Units**	**Minimum**	**Maximum**	**Average ±SD**	**BIS Standards (Max. allowable limit)**	**WHO Standards (Max. allowable limit)**	**Wells nos. exceeding Max. allowable limit**		**Undesirable effect**
							**As per BIS standard**	**As per WHO standards**	
pH	-	7.1	8.2	7.63 ± 0.2134	6.5-8.5	9.2	Nil	Nil	Taste
EC	μmhos/cm	410	3830	1463.48 ± 830.656	-	-	-	-	
TDS	mg/L	267	2345	862.27 ± 494.784	2000	1500	1, 9	1, 2,9, 15, 37	Gastro -intestinal irritation
TH	mg/L	170	1070	441.4 ± 208.396	600	500	1, 2, 6, 9, 10, 15	1, 2, 6, 9–11, 15, 20, 32, 33	Scale formation
T.A	mg/L	155	675	383.6 ± 123.077	600	500	6, 32	2, 6, 9, 14, 15, 24, 32, 37, 39	-
Na^+^	mg/L	0	437	142.37 ± 118.281	-	200	-	1, 2, 9, 10, 15, 24,25, 27, 32, 37	-
K^+^	mg/L	4	76	26.76 ± 20.337	-	200	-	Nil	
Ca^2+^	mg/L	28	188	84.74 ± 29.712	200	200	Nil	Nil	Scale formation
Mg^2+^	mg/L	5	209	55.72 ± 40.976	30	150	1-12, 14–18, 20, 23–25, 31–39, 42, 43	9	Scale formation
Cl^-^	mg/L	28	759	201.76 ± 193.985	1000	600	Nil	1, 2, 9, 15	Salty taste
HCO_3_^-^	mg/L	189	824	468.09 ± 150.209	-	-	-	-	-
NO_3_^-^	mg/L	0	47	7.93 ± 10.452	45	45	1	1	Blue baby
SO_4_^2-^	mg/L	12	300	81.74 ± 65.576	400	400	Nil	Nil	Laxative effect
F^-^	mg/L	0.14	1.5	0.80 ± 0.471	1.0	1.5	4, 8–10, 12, 14–18, 20, 24, 32, 34, 37	Nil	Fluorosis

**Figure 2:**
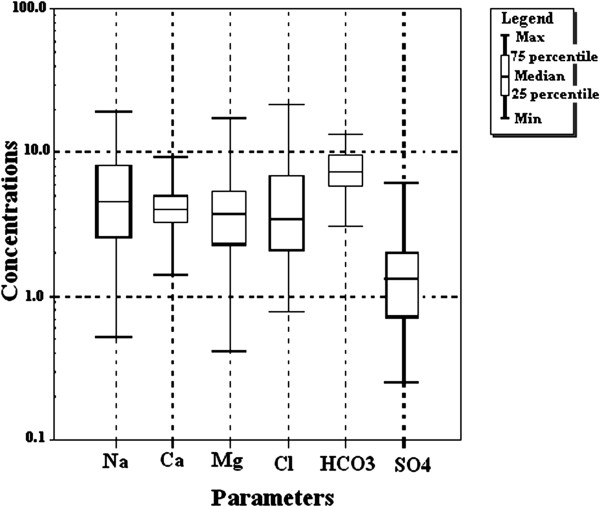
Box-Whisker plot for water quality.

The statistical variation of all the 43 samples have been plotted using Box-Whisker plot (Figure 2), which gives the maximum, minimum and median values for the ionic concentrations. The values of 75 percentile and 25 percentile of various ions can also be obtained through this plot, which gives an idea about the statistical variation of different parameters for the analyzed samples.

### Spatial variation of groundwater quality parameters

**Figure 3:**
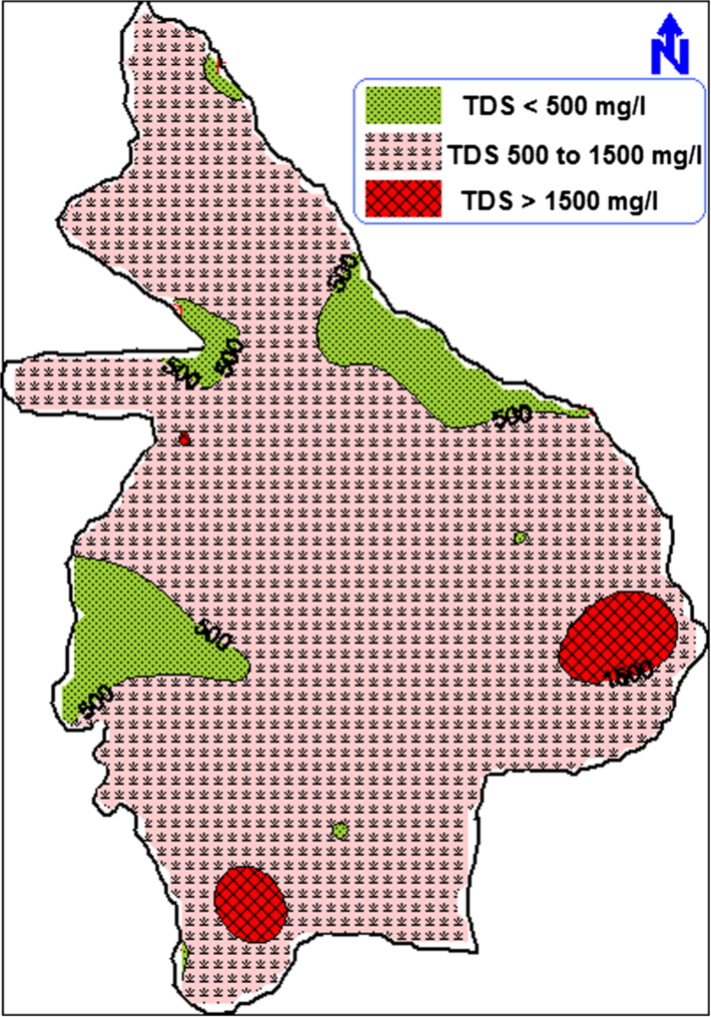
TDS zonation map.

The spatial variation of the TDS in groundwater of Erode city was studied by plotting the TDS values of all 43 groundwater samples using Geographical Information System (GIS) (Figure 3). The TDS zonation map was prepared by setting the most desirable (500 mg/L) and maximum allowable (1500 mg/L) limits. To ascertain the suitability of groundwater for any purposes, it is essential to classify the groundwater depending upon their hydrochemical properties based on their TDS values (Caroll, [19]).

**Figure 4:**
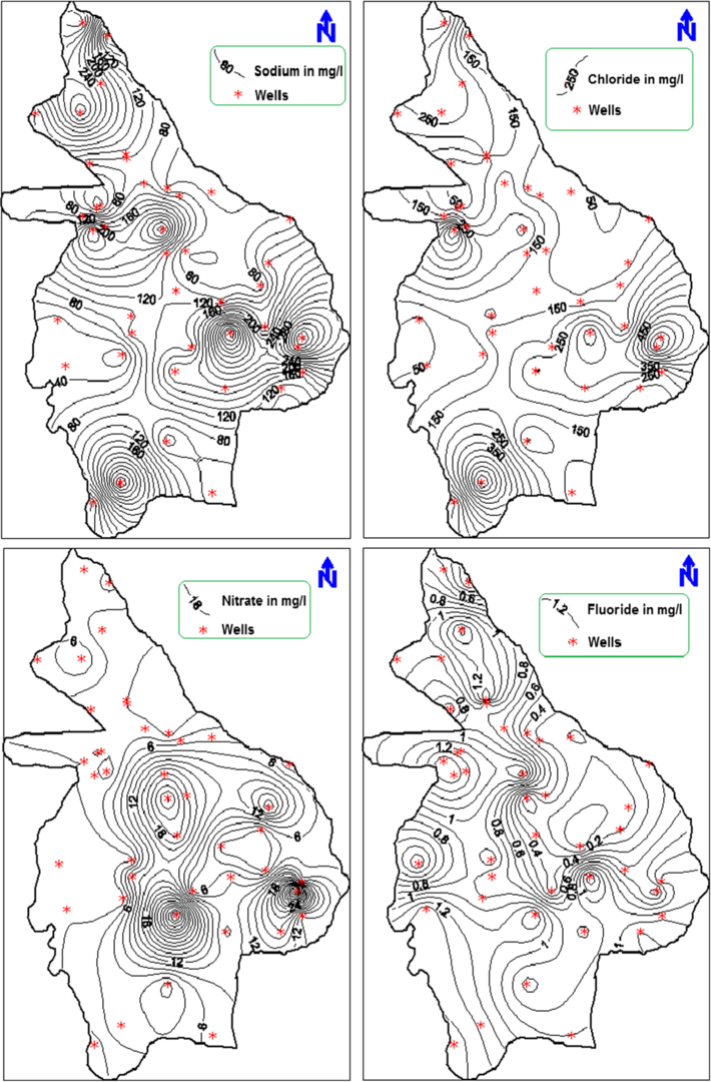
Spatial variation of major ions in groundwater of Erode city.

It was distinctly observed that the groundwater near Vendipalayam landfill site is affected by leachate percolation. The spatial distribution of important geochemical elements like sodium, chloride, nitrate and fluoride were also plotted using GIS (Figure 4). Sodium and chloride ion concentrations exceeded the maximum allowable limit in same locations leading the groundwater unsuitable for drinking purposes. Nitrate and fluoride concentrations were also comparatively high in the areas near Vendipalayam landfill site. Hence, the spatial variation diagrams indicate that the concentration of major ions increased towards east due to the effect of municipal solid waste disposal at Vendipalayam.

### Heavy metals

All 43 groundwater samples were analyzed for heavy metals such as Cu, Fe and Zn, which are characterized as undesirable metals in drinking water. The permissible limits of these metals in drinking water are respectively 1, 0.3 and 5 mg/L (WHO, [17]). Fe concentration in the groundwater samples varied from 0.02 to 0.39 mg/L, and Zn concentration varied from 0.011 to 0.11 mg/L. However, Cu concentration was below detectable limit in all of the samples. Higher concentration of Fe in some pockets may be resulted from the leachate through percolation.

## Discussion

### Leachate properties

The high values of TDS in leachate samples indicate the presence of inorganic materials in the samples. The high BOD and COD values indicate the high organic strength in the leachate of all landfill sites. The presence of ammonia nitrogen is probably due to the deamination of amino acids during the decomposition of organic compounds. The dark brown color of the leachate is mainly attributed to the oxidation of ferrous to ferric form and the formation of ferric hydroxide colloids and complexes with fulvic/ humic substance (Chu *et al.,*[20]). This may be due to disposal of steel scraps into the landfill sites. The presence of trace amount of Pb indicates the disposal of Pb batteries, chemicals used for photograph processing, Pb-based paints and pipes at the landfill sites. The concentration of the contaminants are comparitively lower at Vairapalayam landfill site. This may be due to the presence of river water near the landfill site, which may dilute the leachate concentration.

### Groundwater quality

The groundwater of the area in general is fresh except few places near the landfill sites, which are brackish in nature. Human health is remarkably dependent upon safe and clean drinking water. Preserving the water resources and hindering them from pollution is preferred to the treatment of polluted water and rendering it suitable for consumption (Malakootian and Dowlatshahi, [21]). The high electrical conductivity values obtained in the groundwater samples near the landfill sites are an indication of the effect of leachate on the groundwater quality. The high concentrations of dissolved solids in the groundwater may decrease the palatability and may cause gastro-intestinal irritation in human and laxative effect particularly upon transits (WHO, [17]).

**Figure 5:**
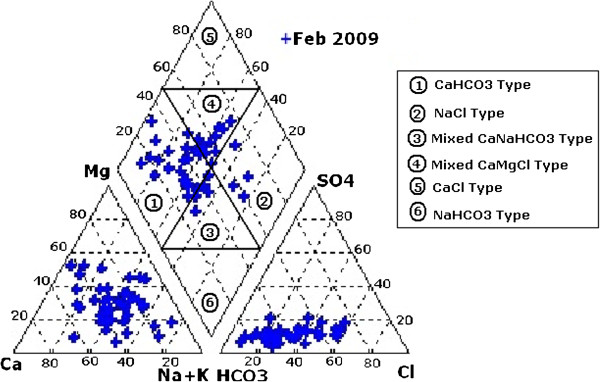
Piper Trilinear Diagram indicating groundwater faciess.

To understand the geochemical evolution of groundwater, the concentrations of major ions have been plotted using Piper trilinear diagram (Piper, [22]) (Figure 5). The percentage reacting values of the cations and anions are plotted as a single point (according to trilinear coordinates) at the lower left and right triangles, respectively. The plot indicates that most of the groundwater samples represent CaHCO_3_ type of water. Some samples fall in the field of mixed Ca-Mg-Cl. Very few samples represent NaCl and mixed CaNaHCO_3_ facies. The sodium absorption ratio (SAR) of the samples also influences the rate of infiltration of water in the soil (Raihan and Alams, [23]).

**Figure 6:**
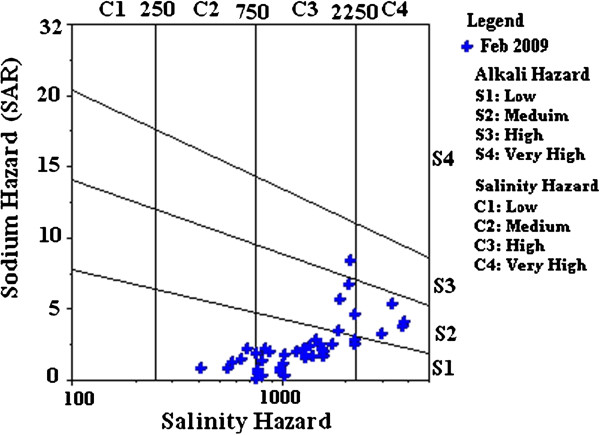
Diagram representing salinity and alkalinity hazard of groundwater.

The analytical results of all the 43 groundwater samples have been plotted in the US salinity diagram (Richard, [24]) to understand the suitability of water for irrigation purposes (Figure 6). Majority of the groundwater samples of Erode city fall in the low sodium class (S1) in the US salinity diagram, which implies no alkali hazard is anticipated to the crops if the water is used for irrigation purpose (Subramani *et al.,*[25]). Few samples represent medium alkali hazard (S2) and only one sample represents high alkali (S3) hazard. However, most of the samples fall in the field of C3 indicateing high salinity hazard. Therefore, the groundwater of Erode city can be classified as high saline and low sodium water which can be used for irrigation purposes on almost all types of soil with little danger of exchangeable sodium (Subramani *et al.,*[25]).

Presence of Fe in groundwater is identified which can lead to change of color of groundwater (Rowe *et al.,*[26]). The concentration of Fe was found well above the permissible limit for drinking as per WHO standard in the samples near to the landfill sites. This shows the influence of leachate on groundwater sources near the landfill sites of Erode city. The metals Pb, Cd, Cr and Ni are characterized as toxic one for drinking water. The migration of metals is likely a product of some parameters including soil sorption capacity, reaction rate of these elements with solid phase, water movement rate in the soil and their primary concentration (Behbahaninia *et al*., [27]). The concentration of these metals, however, was found to be below detection limits in groundwater samples of the Erode city. Even though there may be migration of contaminants into groundwater, the level was below detectable range. This likely indicates that these metals may be adsorbed by the soil strata or by the organic matter in soil. The leachate is generally a strong reducing liquid formed under methanogenic conditions and on coming into contact with aquifer materials has the ability to reduce sorbed heavy metals in the aquifer matrix. The most important reactions are the reduction of Fe and Mn to more soluble species. Hence the concentration of these components increases under favorable conditions close to a landfill and may lead to a serious toxic risk.

### Effect of distance on groundwater contaminations

The extent of contamination level of groundwater quality due to leachate percolation depends upon a number of factors like chemical composition of leachate, rainfall, depth and distance of the well from the pollution source (the landfill site in the present case). Groundwater samples of different depths and distances from landfill sites were analyzed in the present study to understand the level of combination (Mor et al. [4]). From the analysis, it is evident that the concentrations of contaminants were found to be high in the sampling sites which are near to the landfills. Interestingly, the groundwater contamination drops fast with increase in the distance of sampling sites from the landfill sites. The percolation of leachate was further found to become gentler. However, this aspect needs further investigations by drilling more wells of varying depths for having a proper correlation between distance and percolation depth.

Although, the concentrations of few contaminants did not exceed drinking water standard even then the groundwater quality represent a significant threat to public health. Strictly speaking one should avoid using groundwater drawn from the wells located in proximity of the waste dumping sites. If this is unavoidable, deeper drilling and frequent analysis of water samples are desirable. Efforts should be made to supply clean water through pipelines from distant sources.

## Conclusion

The moderately high concentrations of EC, TDS, Cl^-^, SO_4_^2-^, NO_3_^-^, Na^+^ and Fe etc. in groundwater were found near landfill which deteriorates its quality for drinking and other domestic purposes. Further, the presence of Cl^-^, NO_3_^-^ and NH_4_^+^ can be used as tracer with relation to leachate percolation (Mor et al. [28]). As there is no natural or other possible reason for high concentration of these pollutants, it can be concluded that leachate has significant impact on groundwater quality in the area near to all the three landfill sites. The quality of the groundwater was found to improve with the increase in depth and distance of the well from the landfill site. Although, the concentrations of few contaminants do not exceed drinking water standard even then the groundwater quality represent a significant threat to public health.

## Competing interests

The authors are interested in Ground water Quality studies and its interpretation techniques using Remote Sensing and GIS. Interested in research related to Study the Aquifer characteristics and its behaviour.

## Authors’ contributions

RN has collected the samples from the field and analysed for its physical and chemical parameters as well as data related to the study area. ST has guided in the field works, interpretation techniques as well as manuscript preparation. EL has guided in the manuscript preparation. All authors read and approved the final manuscript.
